# SomaScan proteomics reveals novel biomarkers in the progression of liver cirrhosis to hepatocellular carcinoma

**DOI:** 10.1186/s12920-026-02398-3

**Published:** 2026-06-01

**Authors:** Lingyu Huang, Junjun Guo, Wei Liu, Shenping Xie, Qiang Yan, Wenjun Pu, Zhipeng Zeng, Liusheng Lai, Baoyao Wang, Yong Dai, Donge Tang, Huaizhou Chen

**Affiliations:** 1 The Organ Transplantation Department of 924th Hospital of Joint Logistic Support Force of PLA, Guilin, Guangxi 541002 China; 2 Clinical Medical Research Center, Shenzhen People’s Hospital (The First Affiliated Hospital, Southern University of Science and Technology; The Second Clinical Medical College, Jinan University), Shenzhen, Guangdong 518020 China; 3 The first affiliated hospital, School of Medicine, Anhui University of Science and Technology, Huainan, Anhui 232001 China

**Keywords:** Hepatocellular carcinoma, Liver cirrhosis, SomaScan proteomics, Serum biomarkers, Bioinformatics

## Abstract

**Background:**

Hepatocellular carcinoma (HCC) typically develops from liver cirrhosis (LC), however early diagnosis is difficult due to a lack of reliable biomarkers. The goal of this study was to use SomaScan proteomics technology to find plasma protein profiles that differentiated LC and HCC from healthy controls in order to develop novel biomarkers for HCC early detection and targeted therapy.

**Methods:**

We used SomaScan technology to evaluate 10,893 plasma proteins from LC, HCC, and normal populations. Differentially expressed proteins (DEPs) were discovered and functionally annotated using HPA, GO/KEGG, and PPI networks. Venn analysis was used to identify DEPs that were expressed in both LC and HCC.

**Results:**

There were 402 DEPs in LC and 389 in HCC, with MAPK signaling and neutrophil extracellular trap generation being the primary dysregulated pathways in LC and HCC, respectively. In addition, 38 co-expressed DEPs (e.g., SSX7, HIP1R, SLC25A18) were discovered in LC and HCC, including 9 previously unknown potential DEPs. PPI network analysis revealed that FLT4 and PDGFA were key drivers of LC progression to HCC. ELISA experiments confirmed that FLT4 and PDGFA are consistently down-regulated in the progression of LC to HCC (*P* < 0.05).

**Conclusions:**

This investigation described the plasma proteomes of LC and HCC and identified FLT4 and PDGFA as possible early screening targets for HCC, establishing a scientific foundation for HCC detection in high-risk LC populations.

**Supplementary Information:**

The online version contains supplementary material available at 10.1186/s12920-026-02398-3.

## Introduction

Liver disease is one of the main causes of death and disability globally, with liver cirrhosis (LC) and hepatocellular carcinoma (HCC) being the most serious. Cirrhosis is a typical pathological process that occurs throughout the evolution of numerous chronic liver illnesses. It is distinguished by significant hepatic necrosis, fibrosis, and the production of regenerating nodules, all of which eventually lead to liver failure [1]. Hepatocellular carcinoma is the third biggest cause of cancer-related fatalities and the sixth most frequent malignancy in the world, with a poor prognosis and a low five-year survival rate [2]. There is a clear link between cirrhosis and hepatocellular carcinoma, and patients with cirrhosis have a much higher chance of acquiring hepatocellular carcinoma, hence cirrhosis is now considered a precancerous lesion of hepatocellular carcinoma [3]. Early identification of hepatocellular carcinoma is difficult due to confounding factors such as the presence of liver inflammation and cirrhosis, which may reduce the predictive validity of current serologic and radiologic surveillance approaches [4]. Conventional biomarkers, such as alpha-fetoprotein (AFP), have low sensitivity and specificity in screening for cirrhosis and hepatocellular carcinoma, limiting their application in early HCC identification [5]. As a result, innovative serum biomarkers for assessing the risk of cirrhosis and hepatocellular carcinoma with improved diagnostic accuracy and lower cost are urgently required in the clinic.

In recent years, proteomics technologies, especially affinity-based high-throughput proteomics, have become important tools for the discovery of disease-related biomarkers. SomaScan technology, developed by SomaLogic, is an advanced nucleic acid aptamer (SOMAmer^®^ Reagents)-based proteomics platform capable of high-throughput and cost-effective measurement of blood protein levels in blood [6]. SomaScan technique is very specific and sensitive, as it targets and captures target proteins with chemically produced SOMAmer reagents and uses fluorescence signals for relative quantification [7, 8]. This method successfully identified NASH diagnostic markers using ultra-deep focused proteome analysis and confirmed their performance in an independent cohort [9]. Furthermore, research using the SomaScan platform found and validated four discriminative models (SomaSignal) linked with NAFLD, each containing 37 proteins and capable of diagnosing high-risk NASH patients. The hybrid model successfully identified high-risk NASH with good diagnostic abilities, with an AUC of 0.93 in the training cohort, 0.84–0.85 in the validation cohort, and a sensitivity of 0.87–0.92 [10]. These findings show that SomaScan technology has a tremendous promise for detecting and confirming biomarkers associated with liver disease.

**Fig. 1:**
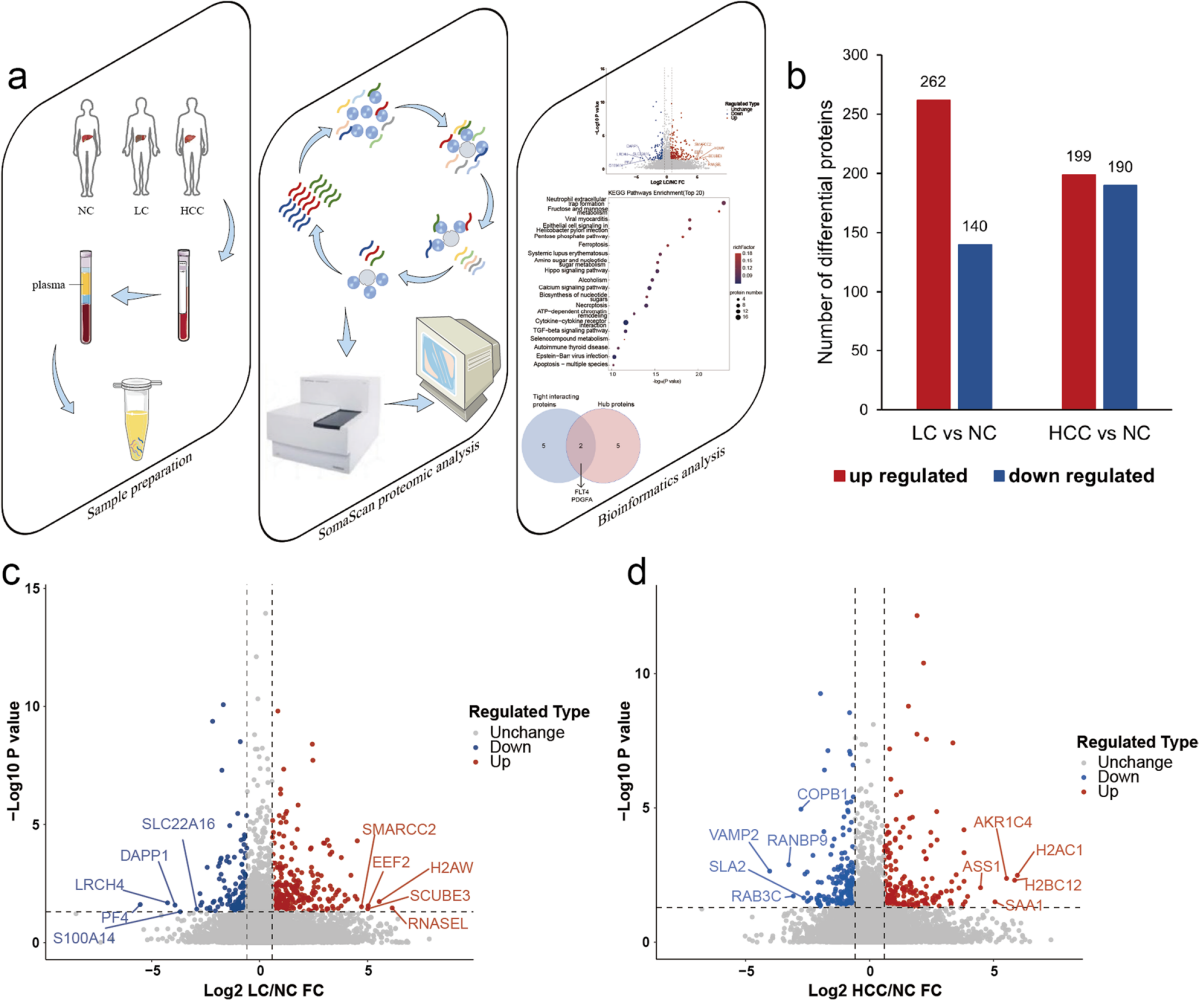
SomaScan proteomics technique identifies serum protein profiles in LC and HCC comparison groups, where NC stands for healthy control, LC for cirrhosis, and HCC for hepatocellular carcinoma. **a** SomaScan proteomics-based analytical technique for investigating serum biomarkers of LC progression to HCC. **b **The total number of differentially expressed proteins (DEPs) found in the LC and HCC comparison groups, with red representing up-regulated proteins and blue representing down-regulated proteins. The volcano figure depicts the distribution of all proteins detected by SomaScan proteomics in the LC (**c**) and HCC (**d**) comparator groups, with red representing up-regulated proteins, blue representing down-regulated proteins, and gray representing proteins that are not significantly expressed

Despite advances in biomarker research, the dynamic proteome alterations that occur during the transition from LC to HCC remain unknown, and current markers (e.g., AFP) are insufficiently sensitive for early-stage HCC. To remedy this gap, the goal of this study was to evaluate plasma samples from LC and HCC using SomaScan proteomics technology, as well as to screen co-expressed proteins that can monitor the progression of LC to HCC carcinogenesis as dual biomarkers using bioinformatics (Fig. 1a). Further integration of PPI network analysis revealed important hub proteins that drive LC-HCC development. To provide potential biomarkers for the diagnosis of early-stage HCC and high-risk LC patients, thereby improving diagnostic accuracy and contributing to a deeper understanding of the mechanisms underlying the progression of LC to HCC.

## Methods

### Sample collection

Based on compliance with all ethical regulations related to this study, 6 patients diagnosed with cirrhosis, 6 patients diagnosed with hepatocellular carcinoma, and 10 healthy controls were collected during their visits to Shenzhen People’s Hospital. All participants signed an informed consent form, and this study was approved by the Ethics Committee of Shenzhen People’s Hospital (LL-KY-2021723) and in accordance with the Helsinki Declaration. Clinical information on patients with LC and HCC is presented in Table 1.

**Table 1:** Clinical data for patients with LC and HCC

Parameter	LC (*n* = 6)	HCC (*n* = 6)	Reference
Age, years	42.17 (7.98)	53.83 (9.34)	18–60
TP, g/L	56.30 (2.09)	60.28 (5.10)	63.00–79.00
ALB, g/L	34.05 (2.22)	36.50 (1.85)	35.00–79.00
GLO, g/L	21.08 (4.08)	25.28 (5.42)	20.00–35.00
A/G	1.74 (0.42)	1.46 (0.36)	1.10–2.50
PA, mg/L	95.17 (45.52)	63.67 (23.75)	150.00-350.00
GLD, U/L	6.60 (2.07)	9.50 (3.48)	< 7.00
TB, µmol/L	268.63 (215.20)	34.18 (11.02)	1.71–20.50
TBA, µmol/L	119.25 (33.00)	99.50 (140.38)	< 10.00
ALT, U/L	97.33 (157.80)	27.17 (12.48)	7.00–55.00
AST, U/L	97.00 (87.23)	54.00 (18.31)	8.00–48.00
GGT, U/L	45.50 (28.03)	111.17 (55.00)	8.00–61.00
ALP, U/L	109.00 (56.09)	127.00 (100.39)	40.00-129.00
CHE, U/L	5677.2 (3888.31)	2185.83 (1145.37)	8k-18k
LDH, U/L	383.17 (283.36)	193.50 (29.68)	140.00-280.00
HBsAg	20744.64 (49744.75)	232.75 (184.34)	N/A
HBsAb	0.56 (0.97)	76.81 (153.46)	N/A
HBeAg	257.24 (628.96)	0.44 (0.38)	N/A
HBeAb	8.18 (18.51)	0.72 (0.73)	N/A
HBcAb	7.00 (3.86)	4.20 (4.64)	N/A
PT, S	26.78 (8.77)	14.00 (2.37)	11.00-13.50
PT%, %	36.5 (17.69)	55.00 (27.90)	100.00
FIB, g/L	1.52 (0.30)	2.10 (0.85)	2.00–4.00
TT, s	25.28 (11.87)	18.37 (2.67)	14.00–19.00
AT Ⅲ, %	33.50 (15.76)	46.67 (26.27)	80.00-130.00
D-DIC, µg/mL	5.45 (6.07)	1.76 (0.64)	0.10–0.25
AFP, ng/mL	17.12 (30.69)	154.30 (50.46)	0.00–7.00
CEA, ng/mL	3.22 (0.86)	37.95 (18.27)	0.00–5.00
Child-Pugh	10.83 (1.94)	6.67 (1.37)	N/A
MELD	4.31 (0.83)	3.84 (1.07)	N/A

Averages are expressed as mean (SD)

*TP* Total protein, *ALB* Albumin, *GLO* Globulin, *A/G* Albumin/globulin ratio, *PA* Prealbumin, *GLD* Glutamate dehydrogenase, *TB* Total bilirubin, *TBA* Total bile acid, *ALT* Alanine transaminase, *AST* Aspartate transaminase, *GGT* Gamma-glutamyl transferase, *ALP* Alkaline phosphatase, *CHE* Cholinesterase, *LDH* Lactate dehydrogenase, *HBsAg* Hepatitis B virus surface antigen, *HBsAb* Hepatitis B virus surface antibody, *HBeAg* Hepatitis B virus e antigen, *HBeAb* Hepatitis B virus e antibody, *HBcAb* Hepatitis B virus core antibody, *PT* Prothrombin time, *FIB* Fibrinogen, *TT* Thrombin time, *AT III* Antithrombin III, *D-DIC* D-dimer, *AFP* Alpha-fetoprotein, *CEA* Carcinoembryonic antigen, *Child-Pugh* Child-Turcotte-Pugh, *MELD* Model of end stage liver disease

a Reference ranges may vary with patient’s sex, age, pregnancy, etc., and may be different depending on materials and methods used.

### PBMC separation

PBMC were extracted from patient peripheral blood via density gradient centrifugation. After centrifugation, the PBMC cell layer was recovered and twice washed with PBS to eliminate any remaining hemocyte components. Cells were counted using a hemocyte counting plate, and cell concentrations were adjusted as necessary for the experiment. To extract proteins, the cells were lysed in RIPA buffer.

### SomaScan proteomic analysis

The SomaScan 11 K assay kit was used in this investigation for proteome analyses. The assay uses somatic protein conjugates (selective single-stranded deoxy oligonucleotides) to quantify proteins based on fluorescence intensity (which indicates relative protein concentration). The SomaScan 11k platform was used to evaluate about 11,000 proteins from plasma samples of NC, LC, and HCC.

Briefly, SOMAmer^®^ reagents were preconjugated to magnetic resin in 96-well plates. Biological samples and controls were diluted in a 1:5 ratio using matrix-specific diluent. Serial dilution was used to create two more dilutions, 1:200 and 1:20,000. The diluted samples were pipetted into their respective 96-well plates. The unbound material was rinsed away following the binding procedure. A biotinylation reagent was applied to each well to identify the proteins on the beads that bind to the SOMAmer reagent. After rinsing away the excess biotinylation reagent, the samples are exposed to UV radiation, which causes the SOMAmer reagent and binding partners to be freed from the magnetic resin. A magnet separates the magnetic resin from the solution before transferring it to a new set of magnetic beads. After binding the biotinylated proteins to the new magnetic beads using the appropriate SOMAmer reagents, a wash is undertaken to remove any SOMAmer reagents that were not coupled to the proteins. Buffer was then added to elute the SOMAmer reagent that had bonded to the protein. A magnet separates the beads and the recovered solution. The eluted SOMAmer reagent is applied to the microarray. During the hybridization reaction, the SOMAmer reagent binds to complementary probes on the array. After washing the microarrays, the slides are processed in an Agilent Microarray Scanning System using a laser to activate the SOMAmer Reagent’s intrinsic fluorophores. The fluorescence intensity is proportional to the number of accessible epitopes on the relevant protein in the original sample. The experimental data is entered into the SomaLogic database, and then the raw data is examined.

### Bioinformatics analysis

Bar graphs were used to illustrate the identified proteins in each sample. The discovered proteins were subjected to volcano plot analysis and HPA annotation with the R package, and differentially expressed proteins (DEPs) co-expressed by LC and HCC were identified using Venn analysis. The DEPs were then evaluated for Kyoto Encyclopedia of Genes and Genomes (KEGG) pathway enrichment and Gene Ontology (GO) annotation enrichment in three categories: biological process (BP), molecular function (MF), and cellular component (CC). Protein-protein interaction (PPI) networks were examined using the Metascape website. In addition, the key proteins of the PPI network were investigated using the Degree approach and the Cytoscape (3.10.1) add-on cytoHubba.

### ELISA validation

In this study, 13 plasma samples were chosen for ELISA validation: 5 from the normal control group, 4 from the LC group, and 4 from the HCC group. Two candidate proteins, FLT4 and PDGFA, were validated. The methods of experiments and data analysis were carried out in line with the manufacturer’s instructions supplied by mlbio.

### Statistical analysis

SomaScan proteomic analysis utilized significanceB to determine P-values, with screening requirements of P-values < 0.05 and |FC|≥1.5. Pearson correlation coefficients were employed for correlation analysis of both DEPs and route enrichment. GraphPad (v10.1.2) was used to analyze data and generate graphs for ELISA studies. All statistical tests were two-sided, with *P* < 0.05 indicating statistical significance.

## Results

### SomaScan proteomic characterization of LC and HCC

SomaScan technology was used in this study to detect the proteome characteristics of LC and HCC. It was able to detect 10,893 proteins expressed in both LC and HCC when compared to the control. We subsequently identified proteins with a 1.5-fold differential fold as differentially expressed proteins (DEPs) and discovered 402 DEPs in LC, including 262 up-regulated DEPs and 140 down-regulated DEPs, and 389 DEPs in HCC, including 199 up-regulated and 190 down-regulated DEPs (Fig. 1b). The identified proteins in LC and HCC were visualized using volcano plots, with blue dots representing down-regulated DEPs and red dots representing up-regulated DEPs (Fig. 1c-d).

### HPA annotation of DEPs in LC and HCC

**Fig. 2:**
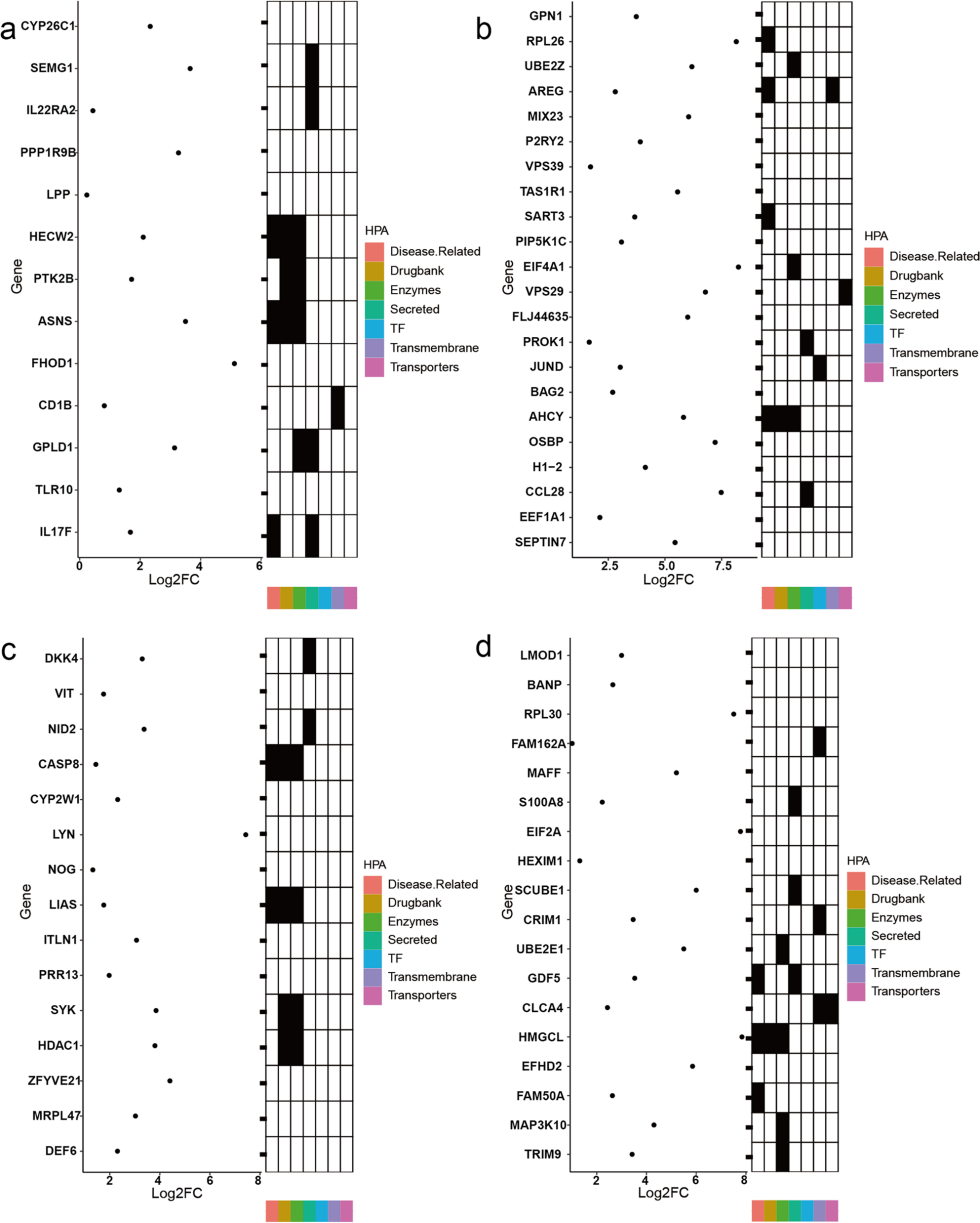
HPA annotations of DEPs. **a** HPA annotations of down-regulated DEPs in the LC vs. NC. **b** HPA annotations of up-regulated DEPs in the LC vs. NC. **c** HPA annotations of down-regulated DEPs in the HCC vs. NC. **d** HPA annotations of up-regulated DEPs in the LC vs. NC

The DEPs were then annotated using the Human Proteome Project (HPA) to investigate their specific types or functions (Supplementary Tables 1–4). Additionally, the top 25 disease-associated proteins were visualized. The results show that among the down-regulated DEPs in LC, HECW2, ASNS, and IL17F are Disease Related proteins, HECW2, PTK2B, and ASNS are Drugbank proteins, HECW2, PTK2B, ASNS, and GPLD1 are Enzymes proteins, SEMG1, IL22RA2, GPLD1, and IL17F are Secreted proteins, and CD1B is a Transporter protein (Fig. 2a). Among the up-regulated DEPs identified by LC, RPL26, AREG, SART3, and AHCY are Disease Related proteins; AHCY is a Drugbank protein; UBE2Z, EIF4A1, and AHCY are Enzymes proteins; PROK1, and CCL28 are Secreted proteins; and JUND is a TF. AREG is a transmembrane protein, while VPS29 is a transporter protein (Fig. 2b).

Among the DEPs down-regulated in HCC, CASP8 and LIAS are Disease Related proteins, CASP8, LIAS, SYK, and HDAC1 are Drugbank and Enzymes proteins, and DKK4 and NID2 are Secreted proteins (Fig. 2c). Among the up-regulated DEPs identified by HCC, GDF5, HMGCL, and FAM50A are Disease Related proteins, HMGCL is a Drugbank protein, UBE2E1, HMGC1, MAP3K10, and TRIM9 are Enzymes proteins, S100AB, SCUBE1, and GDF5 are Secreted proteins, FAM162A, CRIM1, and CLCA4 are Transmembrane proteins, and CLCA4 is a Transporters protein (Fig. 2d).

### GO and KEGG analysis of DEPs

**Fig. 3:**
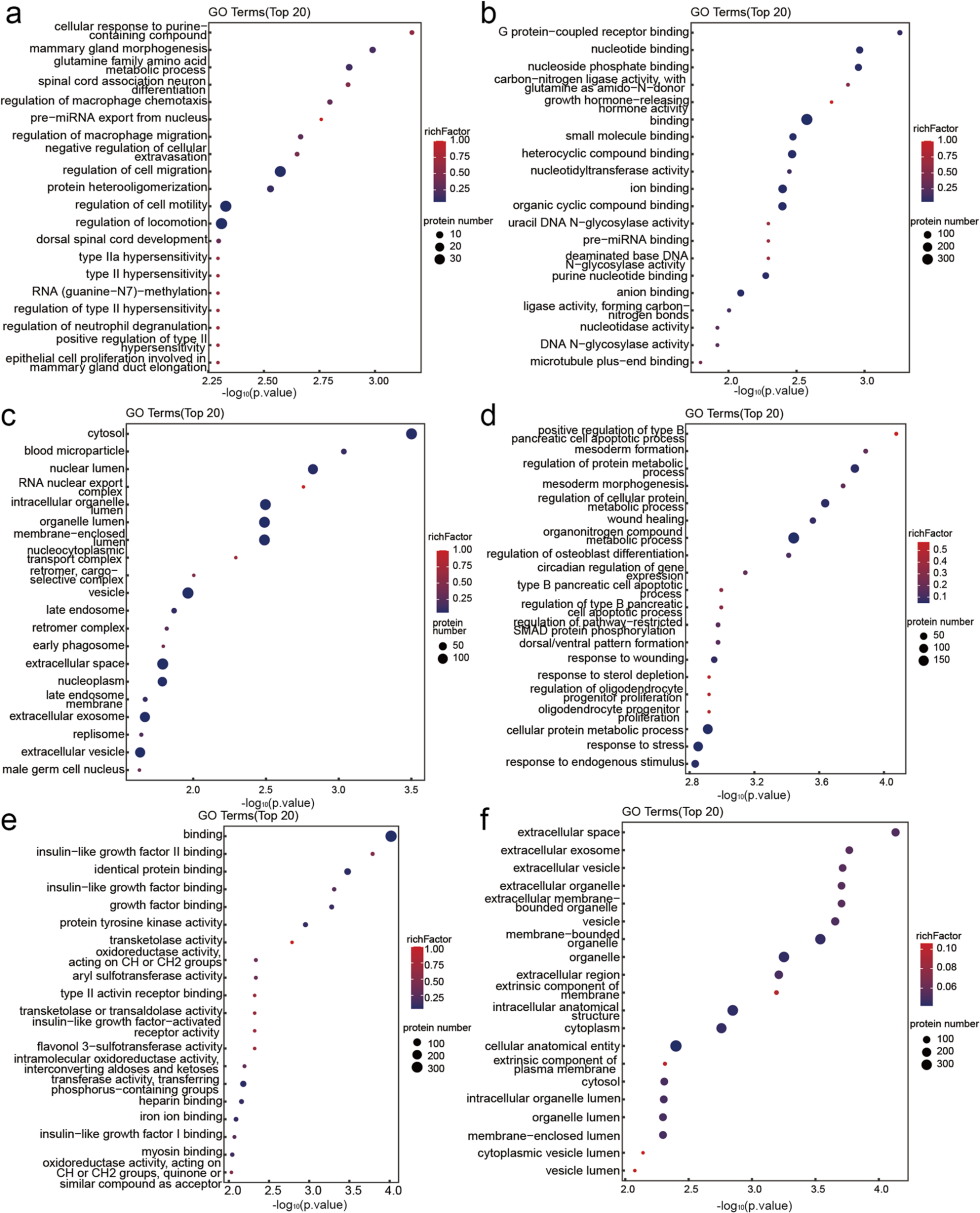
GO functional analysis of DEPs. GO functional analyses of DEPs including BP, MF, and CC were performed for the LC (**a**-**c**) and HCC (**d**-**f**) comparison groups, respectively

**Fig. 4:**
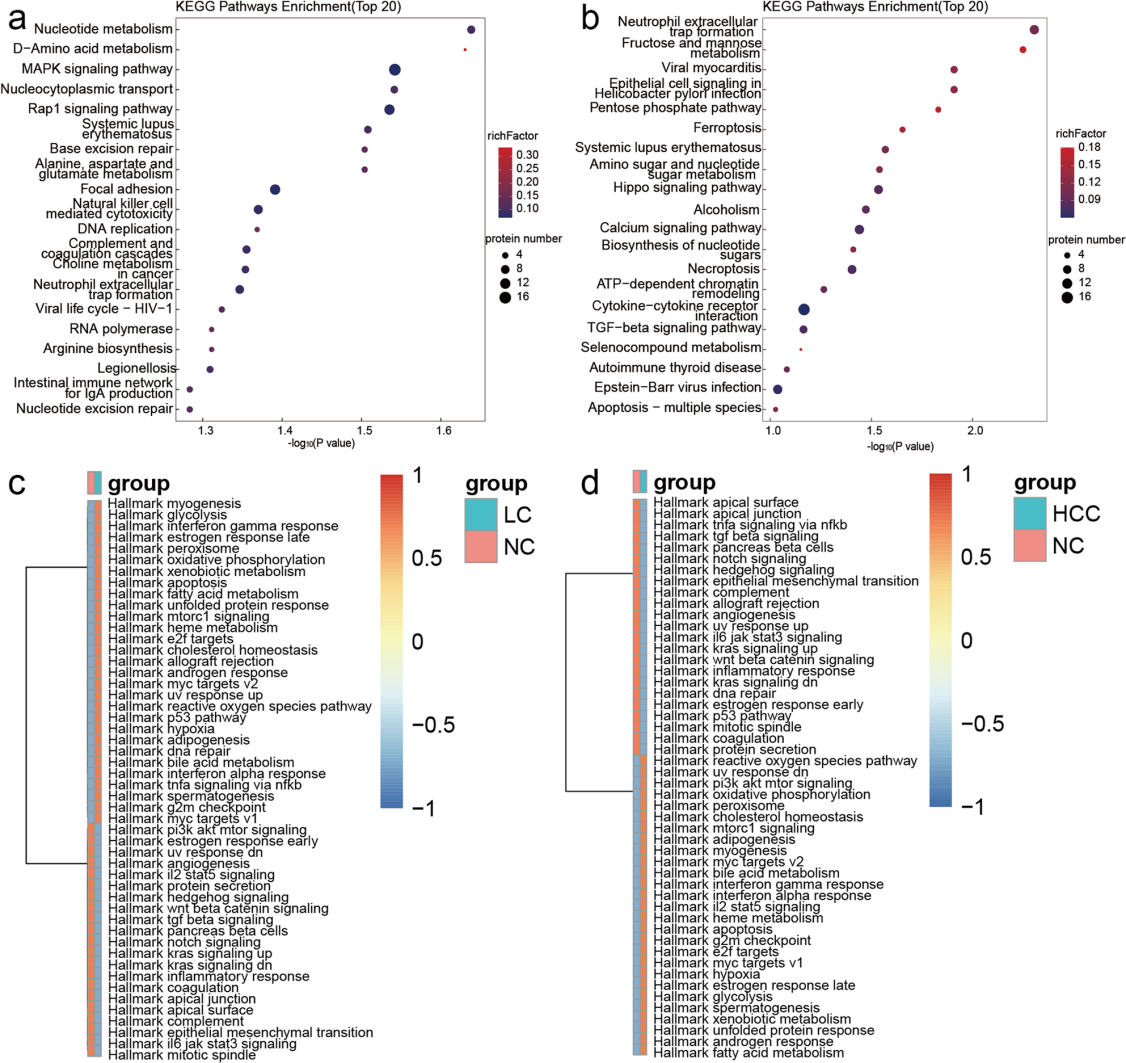
Enrichment analysis of DEPs. KEGG enrichment analysis of DEPs between LC (**a**) and HCC (**b**) comparison groups. ssGSEA enrichment analysis between LC (**c**) and HCC (**d**) comparison groups

The BP, MF, and CC functions of DEPs were determined using GO analysis, and KEGG analysis was used to predict the key pathways of DEPs, with the top 20 significantly enriched results shown. The results showed that DEPs were significantly involved in the cellular response to purine-containing compound, mammary gland morphogenesis, and glutamine family amino acid metabolic process BP function (Fig. 3a); MF results showed that DEPs were associated with G protein-coupled receptor binding, nucleotide binding, and nucleoside phosphate binding functions (Fig. 3b); and the functions associated with CC were primarily cytosol, blood microparticle, and nuclear lumen (Fig. 3c). The KEGG enrichment results analysis revealed that these DEPs were significantly involved in nucleotide metabolism, D-amino acid metabolism, and the MAPK signaling pathway (Fig. 4a).

In the comparison of HCC with NC, DEPs were substantially connected with positive regulation of type B pancreatic cell apoptotic process, mesoderm formation, and regulation of protein metabolic process (Fig. 3d). MF was substantially related to binding, insulin-like growth factor II binding, and identity protein binding (Fig. 3e). In CC, it was substantially related with extracellular space, extracellular exosomes, and extracellular vesicles (Fig. 3f). KEGG enrichment analysis revealed that these DEPs were significantly engaged in Neutrophil extracellular trap formation, Fructose and mannose metabolism, and Epithelial cell signaling in Helicobacter pylori infection (Fig. 4b).

Furthermore, we used ssGSEA to investigate the relationship between molecular function and pathway enrichment in the LC, HCC, and NC comparison groups, and the results were presented as heatmaps (Fig. 4c-d).

### Screening of LC and HCC co-expressed DEPs

**Fig. 5:**
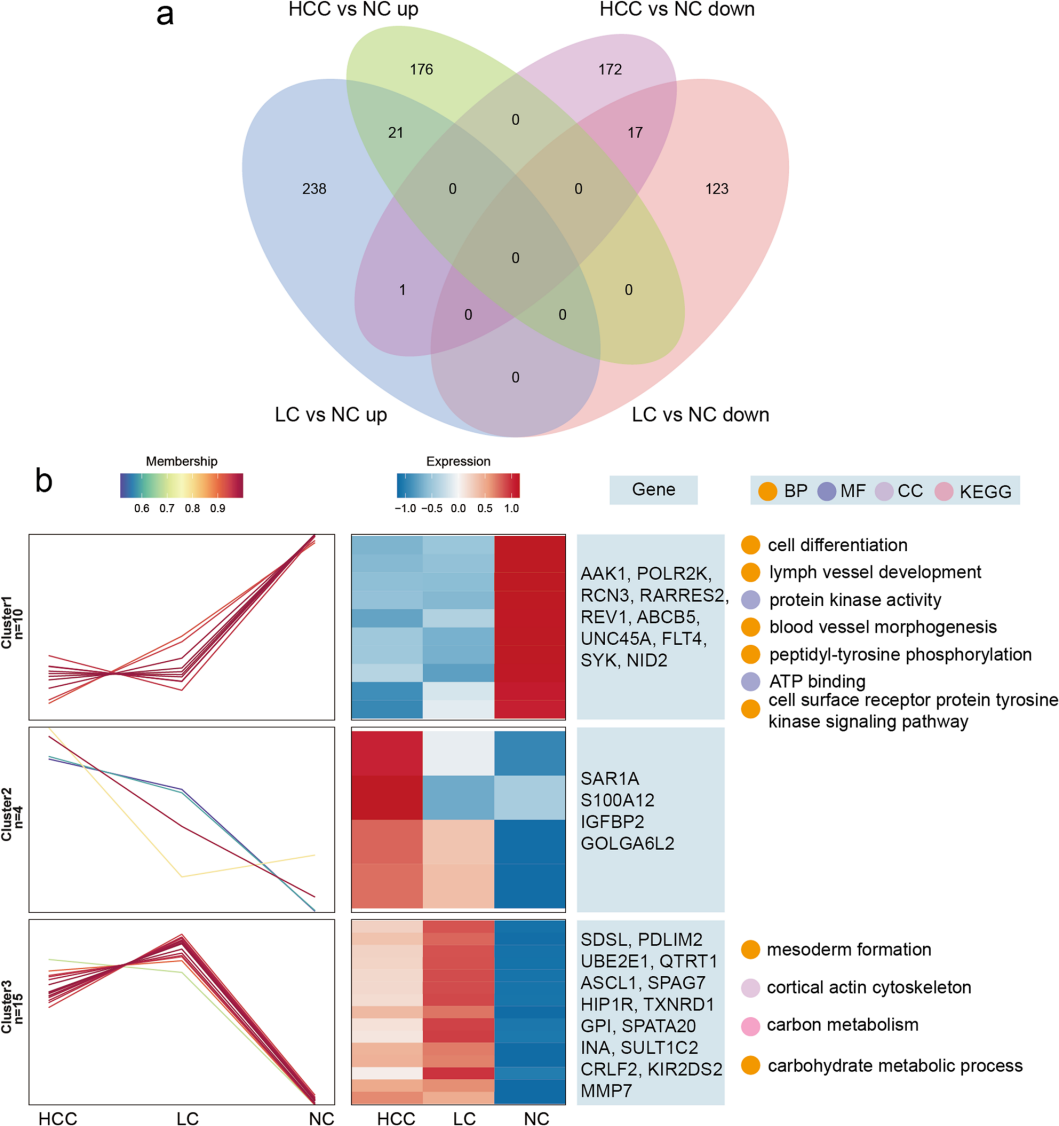
Screening for co-expressed DEPs in LC and HCC. **a** Venn diagram showing co-expressed DEPs in the LC and HCC groups. **b** Expression clustering analysis and functional enrichment analysis of co-expressed DEPs between LC and HCC groups

LC is an important step in the progression of HCC. Exploring the dynamics of co-expressed DEPs in LC and HCC is extremely beneficial for early HCC detection. In this investigation, Venn analysis revealed 38 proteins co-expressed in LC and HCC, including 21 up-regulated and 17 down-regulated DEPs (Fig. 5a). Furthermore, we discovered 9 co-expressed proteins that had not previously been described in LC and HCC through a literature search, including the low-expressed proteins SSX7, VIT, FAM241B, and AAK1 and the high-expressed proteins SPAG7, HIP1R, SPATA20, GOLGA6L2, and SLC25A18 (Table 2).

**Table 2:** Unreported DEPs in LC and HCC compared to NC

NO.	Protein Name	Gene Symbol	LC	HCC	NC	LC/NC	LC/NC *P* value	HCC/NC	HCC/NC *P* value
1	Protein SSX7	SSX7	98.8	97.7	150.4	0.6569149	0.0320374	0.6496011	0.0354884
2	Vitrin	VIT	1443.9	1395.9	2489	0.5801125	0.0341382	0.474086	0.0403068
3	Uncharacterized protein C10orf35	FAM241B	3185.4	3843	6059.9	0.5256522	0.0309015	0.4249461	0.0398838
4	AP2-associated protein kinase 1	AAK1	130.7	134.5	263.9	0.4952634	0.0318158	0.5096628	0.0033214
5	Sperm-associated antigen 7	SPAG7	2654.8	1350.2	509.4	5.2116215	0.0469187	2.6505693	0.0035991
6	Huntingtin-interacting protein 1-related protein	HIP1R	1765.1	955.8	365.3	4.831919	0.0166065	2.6164796	0.0395474
7	Spermatogenesis-associated protein 20	SPATA20	2725	1795	809.7	3.365444	0.000853	2.2168704	0.0499807
8	Golgin subfamily A member 6-like protein 2	GOLGA6L2	2435.8	2947.8	1133.3	2.1492985	5.17E-06	2.6010765	0.0193779
9	Mitochondrial glutamate carrier 2	SLC25A18	458.2	493.7	276.6	1.6565437	0.0465708	1.7848879	0.0112006

*NC* Healthy control, *LC* Liver cirrhosis, *HCC* Hepatocellular carcinoma

### Functional analysis of LC and HCC co-expressed DEPs

LC and HCC co-expressed DEPs were categorized into three gene clusters by expression clustering analysis, and the biological functions of the three gene clusters were further revealed by functional enrichment analysis. As shown in Fig. 5b, the cluster 1 was associated with cell differentiation, lymph vessel development, protein kinase activity, blood vessel morphogenesis, peptidyl-tyrosine phosphorylation ATP binding, cell surface receptor protein tyrosine kinase signaling pathway, positive regulation of MAPK cascade, and positive regulation of protein phosphorylation. phosphorylation biological function related. Cluster 3 is related to mesoderm formation, cortical actin cytoskeleton, carbon metabolism, carbohydrate metabolic process biological function.

### Screening and validation of key proteins associated with the progression of LC to HCC

**Fig. 6:**
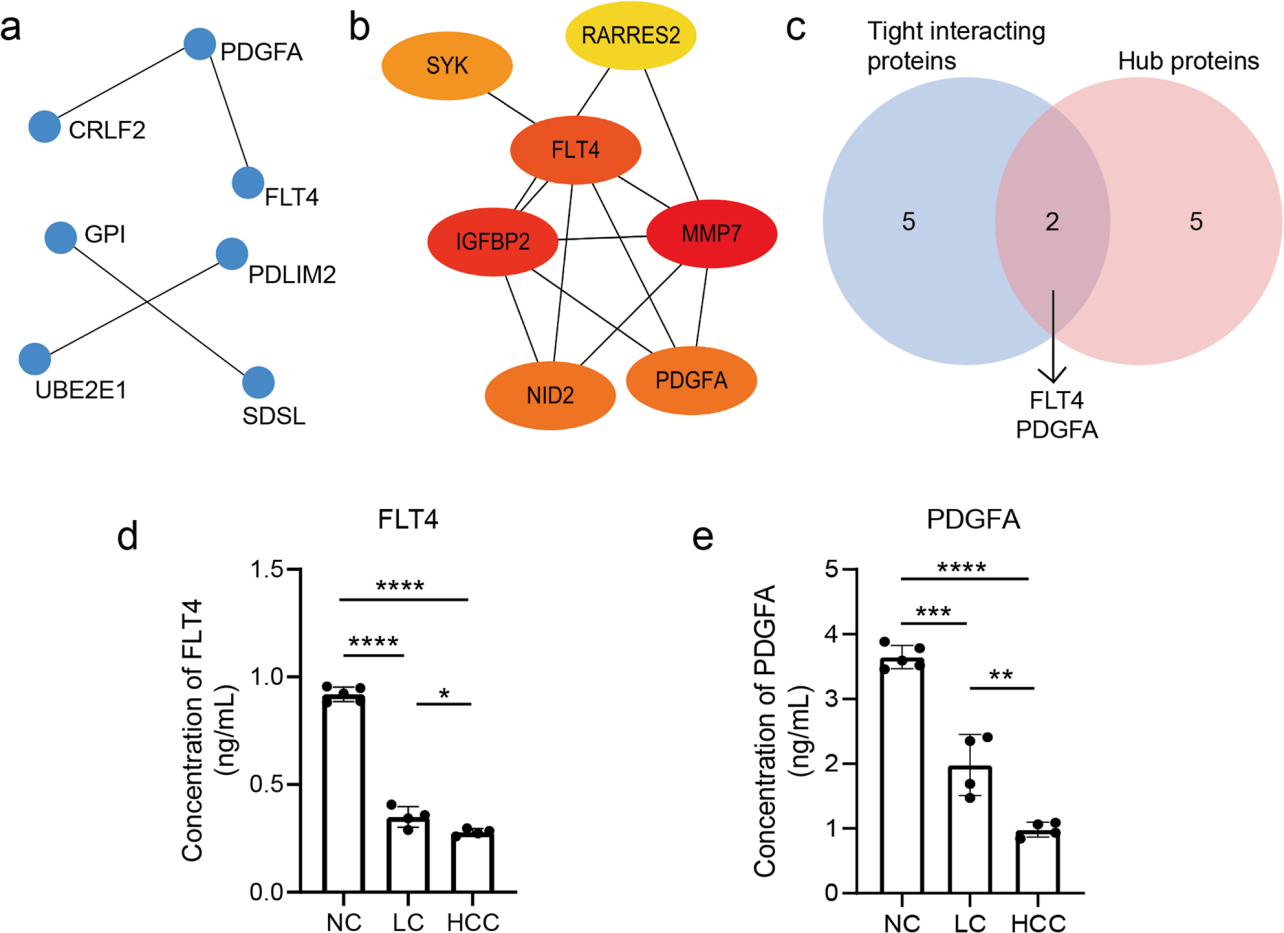
PPI analysis identifies important DEPs for LC development to HCC. **a** Metascape represents the subnetwork of co-expressed DEPs. **b** The cytoHubba algorithm ranks the top 10 Hub proteins among co-expressed DEPs. **c** Venn analysis identifies additional DEPs for LC development to HCC. **d**-**e** ELISA validation of plasma FLT4 and PDGFA proteins

To investigate the relationship between LC and HCC co-expressed DEPs, we used metascape to map the co-expressed DEP subnetwork. As a result, two sub-networks were discovered: CRLF2, PDGFA, and FLT4; and GPI, PDLIM2, UBE2E1, and SDSL (Fig. 6a). In addition, we used the cytoHubba algorithm of the cytoscape software to find the top ten hub genes in the network and calculate the top ten hub proteins, of which seven were linked: SYK, RARRES2, FLT4, IGFBP2, MMP7, NID2, and PDGFA (Fig. 6b). The key proteins for the progression of LC into HCC were then screened further using Venn analysis. FLT4 and PDGFA were identified as possible critical proteins in the progression of LC to HCC (Fig. 6c). The ELISA assay revealed that both the LC and HCC groups had significantly lower plasma levels of FLT4 and PDGFA compared to the healthy control group (*P* < 0.05), with the HCC group showing significantly lower levels than the LC group (Fig. 6d-e), which consistent with the SomaScan results.

## Discussion

Cirrhosis and hepatocellular carcinoma are the next stages of chronic liver disease, however early detection of HCC in high-risk cirrhotic individuals remains a significant therapeutic problem. Ultrasound and α-fetoprotein (AFP) assays have limited sensitivity and specificity for detecting early HCC [11]. In recent years, blood tests have gained popularity as a noninvasive tumor screening method [12, 13]. In this investigation, we used SomaScan proteomics to uncover non-invasive plasma biomarkers that may distinguish LC and HCC from healthy controls, with an emphasis on exposing proteins that are dynamically regulated as LC progresses toward HCC. Our findings not only define the stage-specific proteome, but also identify novel co-expressed proteins that could serve as possible indicators for early HCC diagnosis in LC patients, potentially disrupting the LC oncogenic transition.

Proteomics, which analyzes protein expression, modification, and interaction in HCC tissues or body fluids, can reveal dynamic functional changes that genomics cannot capture [14, 15], providing a critical foundation for molecular typing, early diagnosis, and therapeutic target discovery in liver cancer. In contrast, SomaScan proteomics is a novel protein detection technology with high throughput (~ 11,000 proteins), sensitivity (fg/mL level), and wide dynamic range (10⁶-fold). It can detect low-abundance functional proteins with a small sample volume (e.g., 30 µL of plasma). In this study, SomaScan proteomic analysis revealed significant changes in protein expression levels throughout LC and HCC development when compared to normal control blood. In LC, 402 DEPs were discovered, including 262 up-regulated DEPs and 140 down-regulated DEPs, while in HCC, 389 DEPs were identified, including 199 up-regulated DEPs and 190 down-regulated. This highlights the molecules’ dynamic changes as the disease progresses. Furthermore, HPA annotations identified disease-related proteins in LC, including HECW2 and ASNS, which are involved with ubiquitination and amino acid metabolism, respectively. The down-regulation of HECW2, an E3 ubiquitin ligase linked to tumor suppression in other malignancies, in LC could indicate an early loss of the regulatory mechanism during carcinogenesis [16]. Similarly, ASNS depletion is associated with metabolic reprogramming in LC, which is a marker of aberrant liver function [17, 18]. Down-regulation of CASP8 in HCC suggests resistance to apoptosis and mitochondrial malfunction, which is consistent with prior studies of caspase-8 inactivation in advanced HCC [19]. Patients with high levels of FAM50A have a poor prognosis, and FAM50A suppresses apoptosis and increases value addition in vivo [20]. It should be emphasized that the functional interpretations presented above are based on the known roles of these proteins in other tissues or cell lines as documented in the available literature. However, because the DEPs in this study were derived from plasma, changes in their expression could be influenced by both local liver lesions and systemic responses; thus, caution should be exercised when directly extrapolating the function of individual proteins to the pathological mechanisms of liver disease.

The GO and KEGG enrichment analyses highlighted the pathway-specific dysfunction. In our study, the MAPK signaling pathway was considerably enriched in LC, whereas neutrophil extracellular trap formation was significantly enriched in HCC. Studies have indicated that the enrichment of the MAPK signaling pathway in LC confirms its well-known involvement in fibrosis and inflammation [21], whereas the creation of neutrophil extracellular traps in HCC is consistent with pro-tumor immune microenvironment remodeling [22]. More research is needed to determine the particular mechanisms underlying the deregulation of the MAPK signaling pathway and neutrophil extracellular trap formation in LC and HCC, respectively.

Intriguingly, this study discovered 38 DEPs common to LC and HCC, including 9 previously unknown proteins (such as SSX7, HIP1R, and SLC25A18), providing new candidate targets for HCC early detection. According to studies, SSX7 is a cancer testis antigen [23], while HIP1R is a relative of huntingtin-interacting protein 1, which may be associated with transformed cells in vivo [24]. The increase of the mitochondrial glutamate carrier SLC25A18 could indicate altered glutamine metabolism, which is a metabolic deficit in HCC [25–27]. However, the particular methods by which these unreported plasma proteins contribute to the evolution of HCC must be validated through biological experiments.

Furthermore, PPI network analysis demonstrated that FLT4 and PDGFA act as hub proteins, connecting LC and HCC. According to the available literature, FLT4, a critical regulator of lymphangiogenesis and vascular remodeling, may offer a pathological underpinning for HCC progression in cirrhosis by encouraging aberrant vascular proliferation and the creation of a fibrotic milieu [28, 29]. In contrast, PDGFA, a key regulator of hepatic stellate cell activation, may accelerate the progression of fibrosis to cancer by increasing pro-inflammatory factor release and epithelial-mesenchymal transition [30, 31]. As a result, plasma FLT4 and PDGFA may be useful biomarkers for the progression of LC to HCC. To validate these findings, we used the ELISA method to separately quantify FLT4 and PDGFA protein levels. The study discovered that both the LC and HCC groups had considerably lower plasma levels of FLT4 and PDGFA compared to healthy controls (*P* < 0.05), with the HCC group showing much lower levels. This tendency was consistent with the SomaScan results, indicating that these two proteins have strong repeatability as potential biomarkers.

This paper provides a SomaScan-based plasma proteome atlas with certain limitations, such as the sequencing technology’s singularity, a small sample size, and a lack of functional validation. Biomarker studies based on plasma samples are inherently limited since plasma proteomics reflects systemic changes in the protein profile, making it challenging to differentiate between localized liver diseases and systemic responses in distant organs. We did not apply multiple comparisons correction to the p-values, which could have resulted in the exclusion of certain proteins with real differences; also, the validation proteins were chosen subjectively. Our future effort will incorporate multi-omics data and validate potential proteins through additional biological studies. Furthermore, mechanistic investigations are required to investigate the role of new DEPs such as HIP1R and SLC25A18 in HCC.

## Conclusion

In conclusion, our study used SomaScan proteome analysis to identify possible biomarkers for the progression of LC to HCC. Plasma proteome FLT4 and PDGFA were identified as potential cancer-specific biomarkers for the progression of LC to HCC, and our findings provide a scientific foundation for the early detection of HCC. However, the proposed functional hypothesis still requires further experimental verification.

## Supplementary Information

Supplementary Material 1

Supplementary Material 2

Supplementary Material 3

Supplementary Material 4

## Data Availability

The dataset(s) supporting the conclusions of this article is(are) available in the Science Data Bank repository, DOI: 10.57760/sciencedb.31419.
